# Characterization of the Regulatory Region of the Zebrafish *Prep1.1* Gene: Analogies to the Promoter of the Human *PREP1*

**DOI:** 10.1371/journal.pone.0015047

**Published:** 2010-12-22

**Authors:** Elisa Bernardi, Gianluca Deflorian, Federica Pezzinenti, Victor M. Diaz, Marina Mione, Francesco Blasi

**Affiliations:** 1 IFOM-FIRC Institute of Molecular Oncology Foundation, Milan, Italy; 2 Università Vita Salute San Raffaele, Milan, Italy; University of Texas M. D. Anderson Cancer Center, United States of America

## Abstract

Prep1 is a developmentally essential TALE class homeodomain transcription factor. In zebrafish and mouse, Prep1 is already ubiquitously expressed at the earliest stages of development, with important tissue-specific peculiarities. The Prep1 gene in mouse is developmentally essential and has haploinsufficient tumor suppressor activity [1]. We have determined the human Prep1 transcription start site (TSS) by primer extension analysis and identified, within 20 bp, the transcription start region (TSR) of the zebrafish Prep1.1 promoter. The functions of the zebrafish 5′ upstream sequences were analyzed both by transient transfections in Hela Cells and by injection in zebrafish embryos. This analysis revealed a complex promoter with regulatory sequences extending up to −1.8, possibly −5.0 Kb, responsible for tissue specific expression. Moreover, the first intron contains a conserved tissue-specific enhancer both in zebrafish and in human cells. Finally, a two nucleotides mutation of an EGR-1 site, conserved in all species including human and zebrafish and located at a short distance from the TSS, destroyed the promoter activity of the −5.0 Kb promoter. A transgenic fish expressing GFP under the −1.8 Kb zebrafish promoter/enhancer co-expressed GFP and endogenous Prep1.1 during embryonic development. In the adult fish, GFP was expressed in hematopoietic regions like the kidney, in agreement with the essential function of Prep1 in mouse hematopoiesis. Sequence comparison showed conservation from man to fish of the sequences around the TSS, within the first intron enhancer. Moreover, about 40% of the sequences spread throughout the 5 Kbof the zebrafish promoter are concentrated in the −3 to −5 Kb of the human upstream region.

## Introduction

*PREP1* (also known as *pKNOX1*) codes for an homeodomain transcription factor belonging to the TALE (Three Amino acids Loop Extension) class of homeoproteins [2]. It dimerizes with members of the Pbx family of the same protein class and the dimers are able to bind DNA and form ternary complexes with Hox proteins expressed in the anterior region of the CNS. Trimerization of Prep1, Pbx1 and Hoxb1 is important for the expression of several genes like, among others, *Hoxb1* itself, *Hoxb2*, *Hoxa2* and *Hoxa3* [3], [4], [5], [6], [7], [8].

*Prep1* has important functions in development and in the adult. In mouse, the deletion of the gene induces early embryonic lethality [9]. Homozygous hypomorphic mice expressing 2% of the *Prep1* mRNA have a milder phenotype with embryonic lethality at E17.5 affecting 75% of the homozygous embryos, whereas the remaining 25% homozygous are born and live a normal-length life [10]. However, adult homozygous mice show T cell anomalies, are much more sensitive to insulin action and develop tumors at high frequency [11], [12], [1]. High frequency tumors development, together with other data, indicates that *Prep1* is a tumor suppressor gene [1]. Importantly, Prep1 appears to be essential for the hematopoietic stem cells function [13] and in fact the majority of tumors developed by hypomorphic *Prep1* mice are of hematopoietic origin [1].

In zebrafish (*Danio reio*), there are two paralogs of mammalian *Prep1*: *prep1.1* and *prep1.2* [14]. A glutamic acid-rich region is present in the C-terminus of zebrafish Prep1.1 and mammalian Prep1 and Prep2 proteins. However, in zebrafish, this feature is restricted to the C-terminus of Prep1.2. Moreover, *prep1.1* genomic region has an high synteny with that of human Prep1 [15]. Specific morpholinos induce strong down regulation of *prep1.1*, and embryonic lethality. The embryos present early developmental defects in the hindbrain (with no expression of some of the anterior *Hox* genes) and neural crest cells differentiation with subsequent major cranial cartilage abnormalities. Moreover, they also show weak circulation, pericardial edema and other anomalies [14]. *Prep1.2* loss of function, on the other hand, does not affect hindbrain development but changes the identity of the hyoid cartilage and causes the absence of branchial arches 4-7; an effect similar to the lack of retinoic acid. Indeed, *Prep1.2* responds to RA through a 3′-RARE (Retinoic Acid Responsive Element) located in the first intron [15]. No study has addressed so far the response of Prep1 to retinoic acid in the embryo, but in mouse and human teratocarcinoma cells Prep1 expression is not affected by retinoic acid [5], [16]. These data strongly suggest that the two *prep1* genes of zebrafish carry out, non redundantly, many of the functions of mammalian *Prep1.* On the other hand, in zebrafish the gene retaining characteristics more similar to human *Prep1* is *prep1.1*. For this reason we have studied the zebrafish *prep1.1* promoter.

In mouse embryos, *Prep1* is expressed ubiquitously from the oocyte stage, however with major differences among organs [17], [9]. Likewise, in the adult mouse and man *Prep1* is expressed in many organs, but at different levels [10] and our unpublished data]. In zebrafish, *prep1.1* expression is initially ubiquitous but in the course of development its expression domain becomes much more anteriorly pronounced, mainly in the neural area [14]. No information in available on the adult fish.

The importance of *Prep1* in development and cancer prompts a study on the regulation of its expression. Indeed, as a tumor suppressor, *Prep1* may be a target of methylation- and oncogene-induced silencing. Moreover, *Prep1* appears to control apoptosis [18], [19] and possibly other basic cellular functions. We have therefore analyzed the regulatory region of *prep1.1 in vitro* and *in vivo* in zebrafish as the study of the zebrafish promoter may shed light on mammalian Prep1 regulation. The data obtained reveal some similarities with the human promoter and identify an *EGR-1* binding site, conserved also in man and mouse, which is essential for *prep1.1* transient expression in zebrafish embryos. We have also isolated a transgenic zebrafish in which the GFP gene is expressed under the control of an 1.8 Kb *prep1.1* promoter fragment and in which much of the embryonic *prep1.1* expression pattern is recapitulated. In addition to providing information on the expression of *Prep1* in the adult fish, this will be a very important tool in further studies of the expression of the gene and in the search for possible small molecules regulators.

## Results

### Identification of the 5′ transcription start site (TSS) of human *PREP1* and of the transcription start region (TSR) of the zebrafish *prep1.1* genes

**Figure 1:**
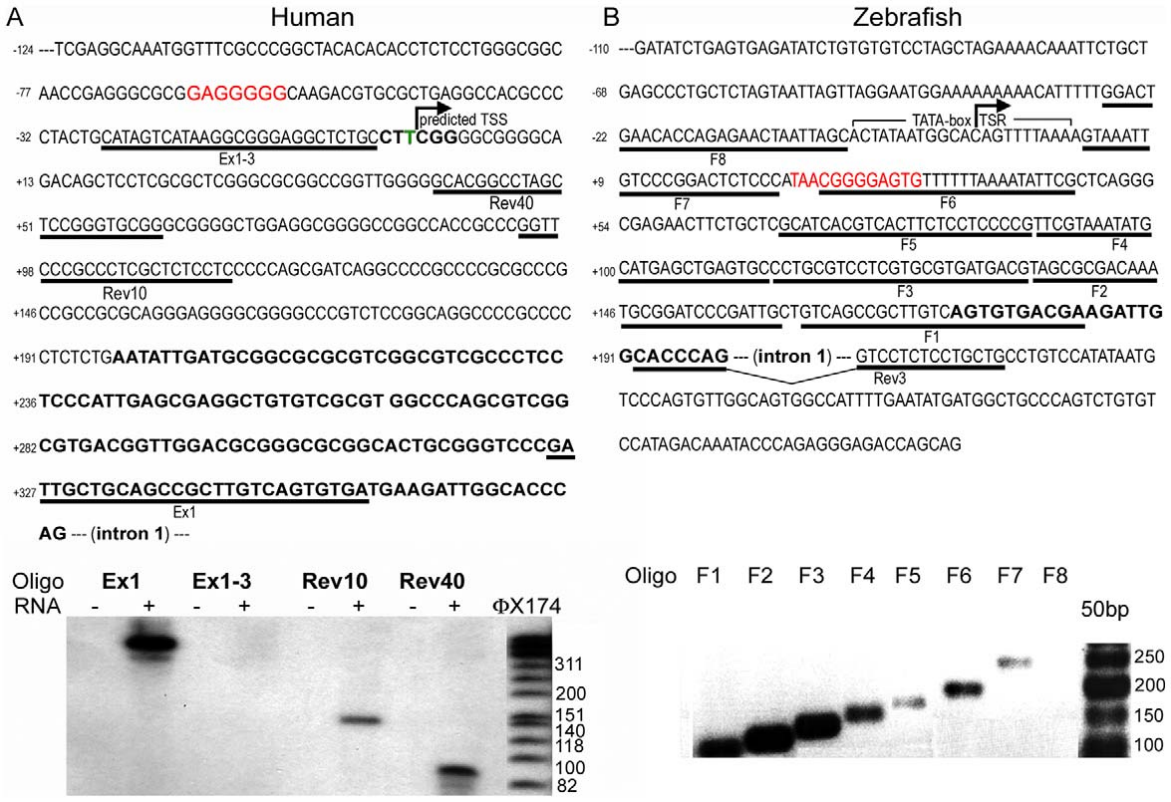
Determination of the Transcription Start Region of human and zebrafish *Prep1* genes. **A**) DNA sequence of the region upstream to the published cDNA sequence (shown in bold). Underlined sequences are the primers employed in the primer extension analysis (Ex1-3, Rev40, Rev10 and Ex-1). The red bold sequence identifies an EGR-1 binding element. The predicted transcription start site (TSS) was identified in this experiment on the basis of the migration of the amplified primers. Below, primer extension analysis using HeLa cells RNA and the primers indicated in A. OX174 lanes show the migration of molecular weight markers (indicated on the side). **B**) DNA sequence of the zebrafish genomic region which includes a predicted TATA box and TSR of *prep1.1* gene. Underlined sequences are the primers used for the RT-PCR assay, F0 to F8 and REV3 (see **Table 1**). The red bold sequence identifies an EGR-1 binding element. Below, agarose gel electrophoresis of the RT-PCR: F7 identifies the last primer able to amplify, together with REV3, the zebrafish cDNA.

We first identified the transcription start site (TSS) of the human *Prep1* gene using a primer extension assay (see Methods). **Figure 1A** (top) shows the DNA sequence upstream of the 5′-UTR region of the published cDNA (which is shown in bold). The figure also shows the oligonucleotides (underlined) which were ^32^P-labeled and used for primer extension (see Methods). HeLa cells total mRNA (50 µg) was primer-extended with reverse transcriptase and the material analyzed on acrylamide-urea gel electrophoresis. **Figure 1A** (bottom) shows that primer Ex1, localized in the 5′-UTR, amplified a band higher than 300 bp. No amplification was obtained with primer Ex1-3 indicating that the TSS was downstream of this sequence. Rev40 amplified a band of 90 bp, setting the TSS 68 bp upstream of this primer. This site corresponds to a thymidine indicated in green in **Figure 1A** (top) and is considered position +1. Primer Rev10, indeed, gives a band of about 140 nucleotides in good agreement with the TSS identified with Rev40**.** As the TSS is determined through the length of the amplified fragment comparing its migration with that of size markers, there can be a minor uncertainty (2-4 nucleotides) on the exact nucleotide from which transcription starts. However, since this uncertainty does not dramatically affect the identification of the upstream regulatory region, we have not investigated this point further. The region does not present an obvious TATA box, but is surrounded by several CpG dinucleotides (**Figure 1A**, top). The sequence is well conserved in the mouse, thus it is likely to be the TSS also in the mouse gene (Figure S1).

To identify the transcription start region (TSR) of the zebrafish *prep1.1* gene, we prepared and retrotranscribed mRNA from zebrafish embryos (24 hpf), and PCR amplified in the 5′ direction and used the sequence corresponding to the 5′-end of the cloned cDNA (accession number NM131891), to design the reverse primer (REV3). As shown in **Figure 1B**, we used different forward primers (F1 to F8) designed on the genomic sequence and coupled them to REV 3 in PCR reactions. The most 5′ primer able to amplify *prep1.1* mRNA was F7 (**Figure 1B**, bottom). Hence, the transcription start region (TSR) is located within the 22 bp of primer F7. The most upstream nucleotide of primer F7 is arbitrarily designed +1.

**Figure 2:**
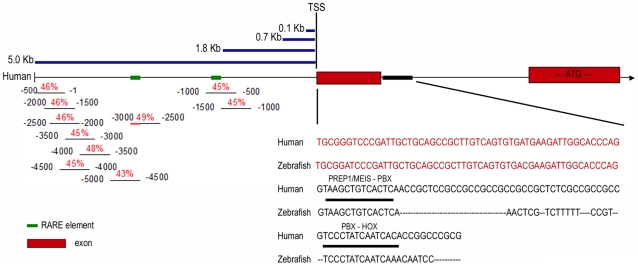
Analysis of the homology between the 5′ and intronic regulatory regions of the zebrafish *prep1.1* and human Prep1. The middle part of the Figure shows a scheme of the 5′ region of human Prep1 gene, including the first two exons (red boxes) and the first intron. The positions of the transcription start site (TSS) and of the translation start sequence (ATG) are indicated. Moreover, the promoter fragments used for the analysis of the Prep1 promoter, measuring 0.1, 0.7, 1.8 and 5.0 Kb, are reported (blue lines). In the bottom left corner there is a schematic representation of the homology in the first 5 kb between the zebrafish and human region, performed blasting 500 bp segments of the zebrafish *prep1.1* regulatory region agaibnst the whole human 5 Kb. The red numbers indicate the percentage of identity, the black numbers show the position of the 500 bp zebrafish promoter segments. The bottom right corner shows the sequence comparison between the first (untranslated) exons (red) and the first 100 bp of the first intron (black) of human and zebrafish Prep1 gene. Prep1/Meis-Pbx and Pbx-Hox binding sites are indicated.

The sequence of the region around the transcription start site of human *Prep1* is conserved in chimpanzee, mouse, fugu *Prep1* and zebrafish *prep1.1* genes. Figure S2 shows that this region is conserved both in the 3′ direction to include most or part of intron 1, and in the 5′ direction. The region of homology to the human sequence is much more extended in chimpanzee (where it includes almost the entire intron 1) than in mouse and even less in zebrafish. The details of the human and zebrafish DNA sequence are reported in **Figure 2** (bottom right section).

### Deletion analysis of the human *PREP1* and zebrafish *prep1.1* promoters in HeLa cells

The sequence conservation around the human TSS (Figure S2) suggests its functional relevance. Moreover, in all species the 5′ end of intron 1 contains potential binding sites for Pbx-Hox and Pbx-Prep/Meis dimers (see the right section of **Figure 2**, Figure S1 and S2 and **Table 1**), suggesting the presence of regulatory elements. Moreover, we have noticed that in mouse and man (but not in zebrafish) the region around the TSS contains several potentially methylatable CpG dinucleotides. Interestingly, 68 bp upstream of the human TSS, an EGR-1 (early growth response-1 transcription factor) binding site (GAGGGGGCA) (Fig. 1A) was identified that is conserved also in mouse (Figure S1). In zebrafish, an EGR-1 binding site is located about 44 upstream of the arbitrarily chosen TSS.

**Table 1:** List of oligonucleotides employed to determine the transcription start site in zebrafish embryos.

	Oligonucleotide
F1	5′-ctgcagccgcttgtcagtgtgacga -3′
F2	5′-tagcgcgacaaatgcggatcccgattg -3′
F3	5′-cctgcgtcctcgtgcgtgatgacg -3′
F4	5′-ttcgtaaatatgcatgagctgagtgc -3′
F5	5′-gcatcacgtcacttctcctccccg -3′
F6	5′-cggggagtgttttttaaaatatttgc -3′
F7	5′-gtaaattgtcccggactctccc -3′
F8	5′-ggacagaacaccagagaactattagc -3′
REV3	5′-cagcaggagaggacctgggtg -3′

All PCRs have been carried out with a single reverse primer (REV3) coupled to an individual forward primer (F). See Figure 2 for the location of the primers in the region upstream of the published 5′ end of the zebrafish *prep1.1* cDNA.

**Figure 3:**
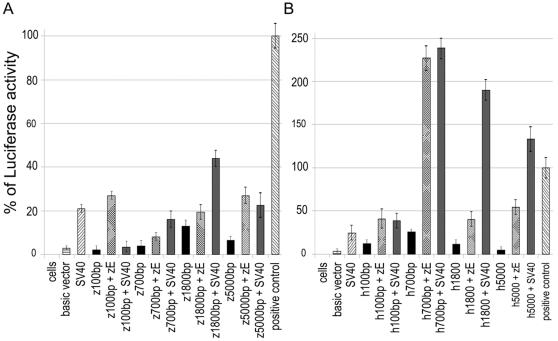
Analysis of *prep1.1* and *Prep1* promoter regions activity in HeLa cells. The graphic data are expressed as relative Luciferase activity (normalized againstb the co-transfected beta-galactosidase), defined as % of luciferase activity. The value of 100% is given to the positive control (SV40 promoter + enhancer). **A**) Activity of the constructs of zebrafish *prep1.1* 5′ regulatory regions. **B**) Activity of the constructs of human Prep1 5′ regulatory regions. *zE*, zebrafish enhancer element (see text).

In order to characterize the human and zebrafish promoters, we prepared four kinds of constructs containing 5.0, 1.8, 0.7 and 0.1 Kb upstream of the TSR from both the zebrafish *prep1.1* and the human *PREP1* gene cloned upstream of a luciferase reporter in the pbasic vector. **Figure 2**, upper left section, shows schematically the cloned fragments. These constructs were transiently transfected in HeLa cells and their luciferase activity measured (see Methods). Moreover, we cloned in the same vectors also the zebrafish intronic sequence (563 bp, *zE*) that contains the Pbx-Hox and the Prep1(Meis1)-Pbx binding sites (right bottom section of **Figure 2** and **Table 2**). As controls, we used the promoter-less pbasic luciferase vector, the SV40 promoter and the full SV40 promoter-enhancer (the activity of which was set to 100% in all experiments). The luciferase activity of the zebrafish promoter (resulting from the average of three independent experiments, each performed in triplicate) are presented in **Figure 3A**. The construct containing the 100 bp upstream of the arbitrary zebrafish TSS (*z100 bp*) has a very low activity, about 15% of the enhancerless SV40 promoter, but is activated about 10-fold when associated to the zebrafish (*zE*) enhancer. Moreover, it is activated, although weakly, also by the SV40 enhancer. These data indicate that the 100 bp fragment containing the *prep1.1* TSR is, in fact, the basal promoter region. The -700 construct (*z700 bp*) also showed low promoter activity (about 20% of SV40), was slightly activated by the zebrafish enhancer, and highly by the SV40 enhancer. The −1800 bp construct (*z1800 bp*) has a stronger basal activity that increases 2-fold in the presence of the SV40 and about 60% in the presence of the zebrafish enhancer. Finally, the −5 Kb construct (z5000 bp) has a basal level which is 30% of SV40 and is activated 4-fold by the zebrafish and SV40 enhancer. These data therefore confirm the functionality of the zebrafish TSS region and indicate that the zE element has enhancer activity.

**Table 2:** Conservation of binding sites for Prep1, Pbx in the first intron of the Prep1 gene.

	Pbx-Hox	Pbx-Prep1 (Pbx-Meis)
**Human**	TGACGGTTGGACGCGC	GCTTGTCAGTG
**Zebrafish**	TGTCAGTGTGACGAA TGACTGAAAGAGAG TGAGGGACAGGAGG	AGCTGTCACT
**Mouse**	TGCTACTGGCGTGAGTGCGTGTGATGA	CTTTGTCAGT

The data obtained with similar constructs of the human *PREP1* promoter (average of three experiments in triplicate) are presented in **Figure 3B**. The h100 bp construct has low but clear promoter activity (half of SV40) and is activated 3-4-fold by the zebrafish and SV40 enhancer. Thus the zE element affects also the human *Prep1* promoter. The h700 bp has a higher basal activity and is activated 10-fold by both enhancers. The h1800 bp construct, however, has a lower basal activity and is activated by both enhancers (less in the case of the zebrafish). Finally, the h5000 bp construct has an even lower basal activity but otherwise behaves as the h1800 bp construct.

Overall these data configure a complex promoter for the *Prep1* gene that spreads for 1.8–5 Kb, and show that upstream sequences regulate transcriptional efficiency. Importantly, the conserved intronic sequence of zebrafish (zE) can have enhancer activities on both the zebrafish and human promoters.

### *prep1.1* promoter fragments direct expression in zebrafish embryos

**Figure 4:**
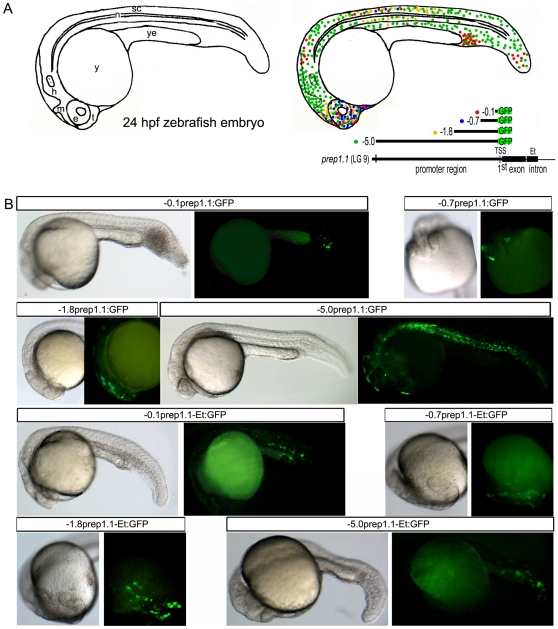
Transient expression of zebrafish *prep1.1:GFP* promoter constructs in 24 hpf zebrafish embryos. **A**) Schematic drawing of a 24 hpf zebrafish embryo, in which the main anatomical features are shown. On the same drawing, the transient expression patterns of all the zebrafish promoter contructs used for the analysis are reported: each dot corresponds to a group of GFP-positive cells, whereas each color is related to different promoter region (see map below). This pattern is the result of dozens of observations (see **Table 3**) manually annotated after each microinjection experiment and stereomicroscope observations. **B**) For each construct (indicated in the stripe at the top of each figure), a representative image of its transient expression *in vivo* is shown. Sometimes the entire embryo is shown (in the lateral view), sometimes only the anterior region (in frontal or lateral view). *e*, eye; *Et*, intronic enhancer; *h*, hindbrain; *m*, midbrain; *n*, notochord; *sc*, spinal chord; *t*, telencephalon; *TSS*, Transcription Start Site; *y*, yolk; *ye*, yolk extension.

The above constructs were also cloned into the GFP-reporter XigDpLynker plasmid and micro-injected into fertilized zebrafish eggs (see Methods). After 24 hours, embryos were observed under a fluorescent microscope and the number of fluorescent embryos and the location of the fluorescence was scored. The data are summarized in **Table 3** and in **Figure 4**. The 100 bp construct(-0.1*prep1.1*:GFP) expressed GFP either in the head and trunk (40%) or in a presumptive (see below) hematopoietic region (60%). The presence of the enhancer (-0.1*prep1.1*-Et:GFP) increased the expression mainly in the head region. The 0.7 kb promoter (-0.7*prep1.1*:GFP) appeared to direct the expression exclusively in the head, both with (-0.7*prep1.1*-Et:GFP) and without the enhancer. The 1.8 Kb construct (-1.8*prep1.1*:GFP) expressed GFP either in the head or in the head and trunk, and in 20% of the latter also in the presumptive hematopoietic region. The presence of the enhancer (-1.8*prep1.1*-Et:GFP) increased mainly the expression in the head. Interestingly the 5 Kb promoter (-5.0*prep1.1*:GFP) was expressed ubiquitously in the absence of the enhancer but exclusively in the presumptive hematopoietic region in the presence of the enhancer (-5.0*prep1.1-Et*:GFP). These data therefore confirm the promoter nature of the constructs and the functionality of the enhancer element, which may have also tissue-specific silencing properties. Expression of the human promoter constructs in zebrafish embryos essentially failed (**Table 3**).

**Table 3:** Deletion analysis of the zebrafish *Prep1.1* and human *Prep1* promoters by injection into fertilized zebrafish oocytes.

Construct	N injected	N GFP^+^	Expression	Expression F1	Expression F2
-*0.1prep1.1:GFP*	210	45	40%: head and trunk60%: hematopoietic region		
-*0.1prep1.1-Et:GFP*	220	48	General increase in expression. In 56% only in the head.		
-*0.7prep1.1:GFP*	207	52	99%: head1% trunk		
-*0.7prep1.1-Et:GFP*	198	52	Increased expression in the head		
-*1.8prep1.1:GFP*	200	49	50%: head50%: head and trunkof which 20% in the hematopoietic region	Ubiquitous,weak	Follows endogenous *Prep1.1* expression
-*1.8prep1.1-Et:GFP*	210	50	Increased expression mainly in the head		
-*5.0prep1.1:GFP*	198	40	100% ubiquitous	Ubiquitous, weak	
-*5.0prep1.1-Et:GFP*	215	46	100% in the hematopoietic region		
-*5.0prep1.1mutEGR:GFP*	161	30	Few cells in the forebrain (60%) and in the notochord (40%)		
h0.1 kB	205	0			
h0.7 kB	187	10	In 9 cases, one or two cells (head); one case, ubiquitous.		
h1.8 kB	168	0			
h5.0 kB	200	0			

A color-coded scheme summarizing the expression data obtained *in vivo* with the various constructs is shown in **Figure 4A**. **Figure 4B** shows some representative examples of GFP fluorescence driven by the various constructs of the *prep1.1* promoter injected in zebrafish embryos. In particular, in the presumptive hematopoietic region with the 0.1 Kb construct, in the head with the 0.7 Kb and 1.8 Kb, and the ubiquitous expression of GFP with the 5 Kb construct. Representative enhancer-containing constructs in **Figure 4B** show GFP expression in the presumptive hematopoietic region (0.1 Kb, panel K), the increased expression in the head (0.7 Kb, panel M and 1.8 Kb, panel N) and the exclusive localization in the presumptive hematopoietic region (5 Kb, panel O). In all cases, expression was obtained in at least 25% of the injected embryos (see **Table 3**).

**Figure 5:**
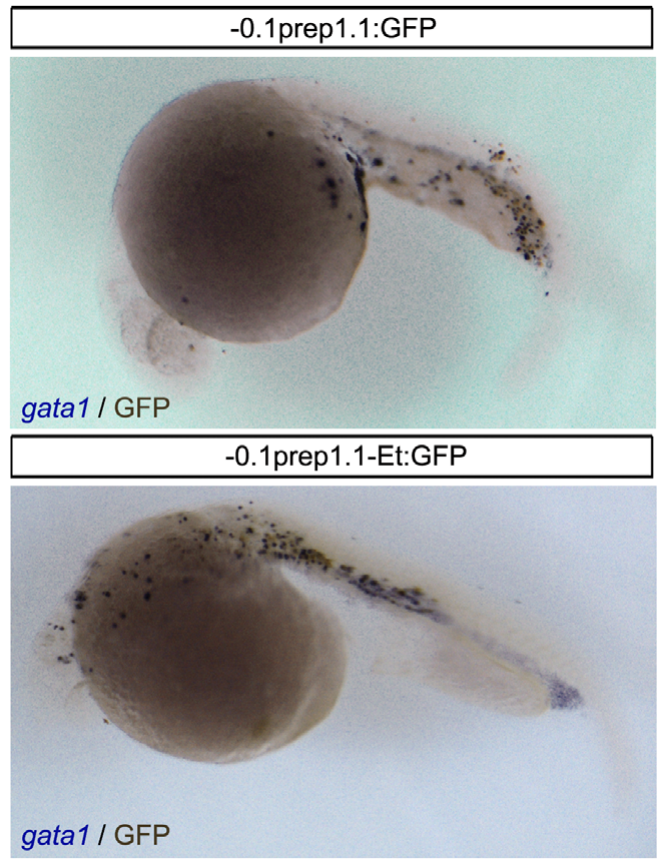
GFP is expressed in some *gata1*-positive cells. Combined *in situ* hybridization for *gata1* (purple) and immunohistochemistry for GFP (dark brown) in 24 hpf embryos injected with the 0.1 Kb promoter constructs (-0.1*prep1.1*:GFP and -0.1*prep1.1*-Et:GFP) reveals the co-localization of the two signals in the most of embryo.

We have confirmed the “presumptive” hematopoietic localization of the 0.1 Kb promoter-enhancer construct (-0.1*prep1.1*-Et:GFP) by performing a combined in situ hybridization for *gata1* and immunohistochemistry with anti-GFP antibody. **Figure 5** shows two examples in which GFP is expressed in the *gata1* positive region.

**Figure 6:**
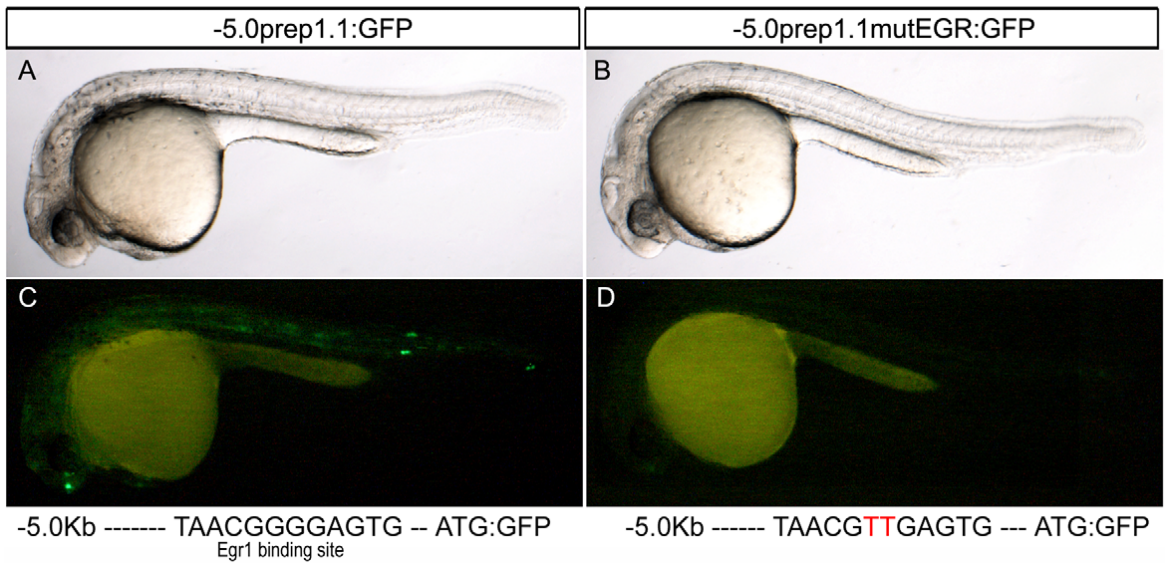
Mutation of the EGR-1 binding site abolishes the GFP transient expression due to the 5.0 Kb construct. The nature of the injected construct is indicated in the stripes at the top of each figure (-5.0*prep1.1*:GFP or -5.0*prep1.1*mutEGR:GFP). Lateral views of 30 hpf embryos are shown. Below each figure, the sequence of the wild type or of the mutated (in red) EGR-1 binding site is reported. In the most of cases (see also **Table 4**) the mutation causes the complete abolishment of GFP expression.

We next tested the role of the conserved EGR-1 binding site (TAACGGGGAGTG) of the *prep1.1* promoter (Fig. 1A **)**. We introduced a mutation in the EGR-1 site (GGGG to GTTG) into the -5.0 Kb construct (-5.0*prep1.1*mutEGR-1:GFP) and injected it into zebrafish embryos. **Figure 6** shows that the embryos injected with this construct essentially did not express GFP. Number of injections and of expressing embryos are reported in detail in **Table 3**. This experiment proves the relevance of the sequence immediately preceding the TSS in *prep1.1* promoter and suggests that *in vivo* EGR-1 may be required for the expression of *Prep1.1*.

### A transgenic fish expressing GFP under the -1.8 Kb promoter has an expression pattern overlapping with that of endogenous *prep1.1*

**Figure 7:**
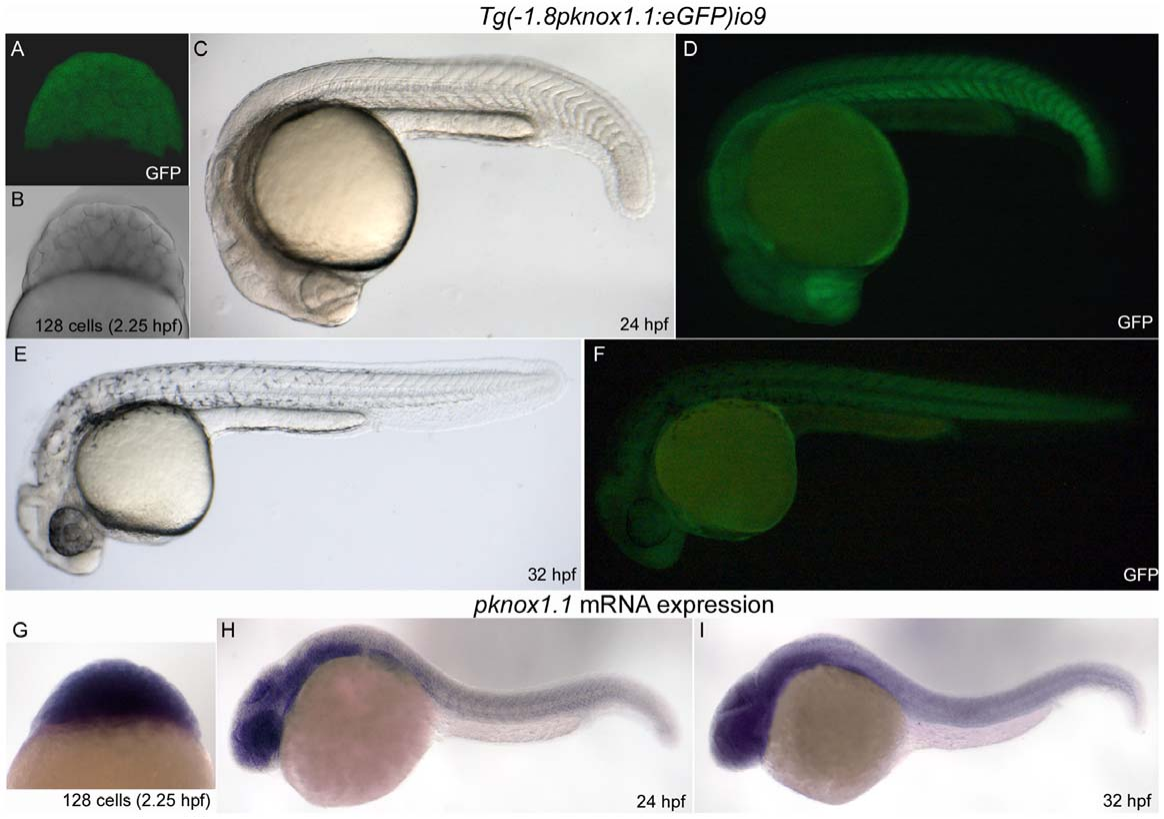
A transgenic fish expressing GFP under the 1.8 Kb zebrafish *prep1.1* promoter recapitulates the expression of the endogenous gene during embryogenesis. A–F: live *Tg(-1.8pknox1.1:eGFP)io009* embryos of three different developmental stages (indicated) visualized by confocal (A) or fluorescence microscopy (D, F). Embryos in B, C and E are the same of A, D and F respectively, visualized in transmission (A) and bright field (C, E). G, H, I: In situ hybridization of embryos at the same stage of development with the *pknox1.1* antisense RNA-probe, shown in lateral view.

Embryos injected with the various constructs were cultured and bred in order to obtain germ-line transmission. In two of the embryos the transgene (-1.8*prep1.1*:GFP) integrated into the germ line but an F2 line could be derived only from one of them. In this line, we compared the expression of GFP in live embryos and that of endogenous *Prep1.1* by in situ hybridization (ISH) using an antisense *prep1.1* probe. The results are reported in **Figure 7**, that shows confocal fluorescence of 128-cells embryos (**Figure 7A**) and transversal transmission microscopy (**Figure 7D** and **7F**) in 24 and 32 hpf embryos. Immunohistochemistry result is shown in **Figures 7G, 7H** and **7I**. The two techniques largely identify the same regions in particular in the anterior areas. We conclude that the 1.8 Kb construct reproduces the expression of *prep1.1* fairly faithfully during the first three days of development. In particular, it reproduces the initial ubiquitous expression of the first hours and the subsequent focusing to the anterior region [14].

**Figure 8:**
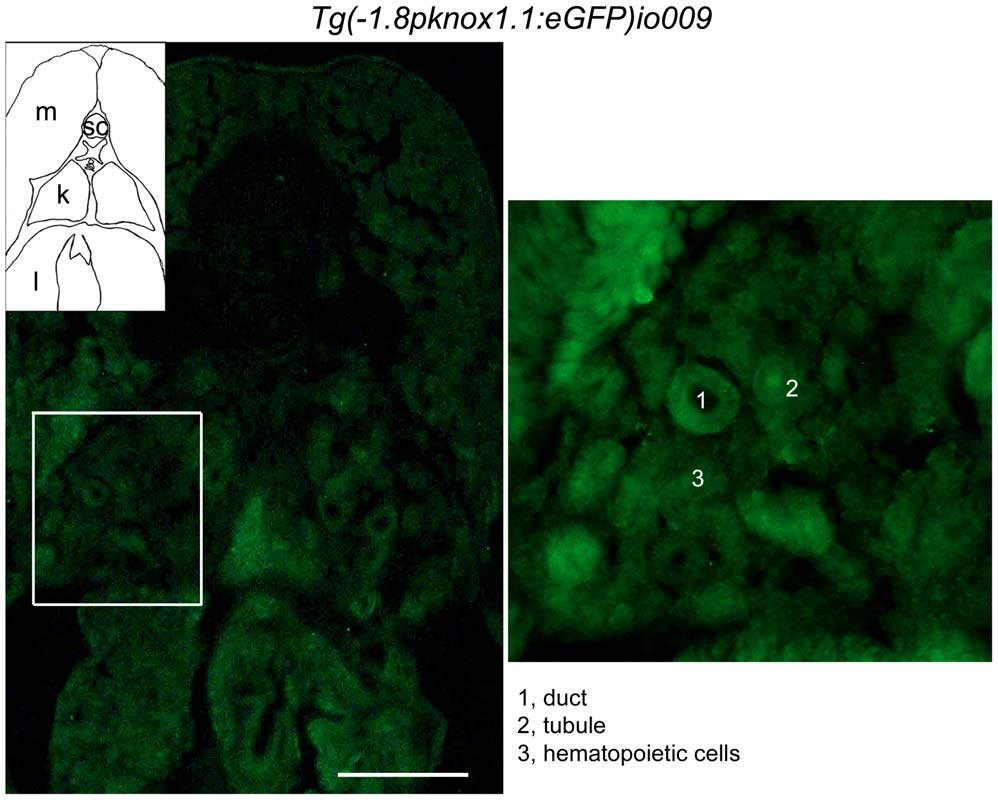
In 6 weeks old *prep1.1*:GFP transgenic fish, the GFP is expressed also in the hematopoietic regions. Cross section, at the level of the trunk, of a juvenile transgenic fish (6 weeks old). A strong expression of GFP was observed in the liver and kidney (the hematopoietic organ in the adult). On the right, the outlined area has been magnified four times and ducts, tubules and hematopoietic cells are indicated. Scale bar: 50 µm.

The expression of *prep1.1* in the hematopoietic area was also confirmed in the adult transgenic fish. Figure 8 presents a transversal cryosection of the trunk showing the GFP fluorescence of kidney and liver areas of a 6 weeks-old fish, i.e. of the hematopoietic areas. The magnified region identifies ducts, tubules and hematopoietic cells from the kidney.

GFP fluorescence was also obnserved in the brain, muscles, pharynx and intestine (not shown). These data agree with the ubiquitous expression of Prep1 in the adult mouse [17], [13].

### Sequences spread through the 5 Kb of the *prep1.1* regulatory region are conserved within −3 to −5 Kb of the human 5′ upstream region

We also analyzed whether zebrafish and homo sapiens share homologous sequences in the 5′ upstream region but found no apparent sequence conservation. We therefore subdivided the 5 Kb zebrafish region into 500 bp segments and compared each of them with the entire human 5 Kb. We found that each zebrafish segments was about 40% identical to sequences present within the region from −3 Kb to −5 Kb of homo sapiens (particularly in the last 1000 bp) (**Figure 2**, left bottom section). Overall these comparisons indicate that several features of the *Prep1* regulatory region are conserved and hence identify potential common regulatory sequences.

## Discussion

Primer extension analysis has restricted the TSS of human *PREP1* and zebrafish *prep1.1* to a 2-4 nucleotides-long region in man and to a 20 nucleotides-long region in zebrafish. These regions are conserved between mammals and fishes.

Deletion analysis in Hela cells of the 5′ regulatory region shows that the presumptive TSS has indeed promoter activity which is regulated by upstream sequences and by an enhancer located in the first intron. In HeLa cells, expression of the zebrafish promoter increased with the length of the inserted sequence between −100 and −1800, but then strongly decayed in the −5000 bp construct, indicating the presence of a negative regulatory element between −1800 and −5000. The presence of the intronic enhancer however bypassed this negative element. In human constructs, the activity was largely restricted to the first 700 bp and decreased in larger constructs. However, the intronic zebrafish enhancer strongly stimulated the activity of all larger constructs (**Figure 3**).

Thus, although artificial, the HeLa cells assays suggest a cooperation of the promoter with distant enhancer regions in the expression of the gene.

**Figure 9:**
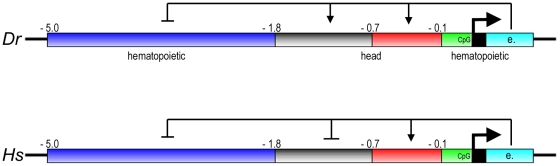
Schematic representation of the zebrafish (Dr) and human (Hs) prep1.1 promoter regions. The tissue-specificity of zebrafish subregions is indicated. CpG identifies a CpG-rich region. “e” indicates the first exon.

Upon injection in zebrafish embryos, the various constructs are able to direct the expression of GFP to specific areas. In particular, the shortest sequence drives expression in the hematopoietic region, whereas sequences between −100 and −1800 nucleotides direct the expression mostly to the head and trunk, and additional sequences appear to have ubiquitous expression properties. Also in this case the conserved enhancer sequence (zE) appears to have regulatory activity, which may be tissue-specific since its presence often modifies the domain of expression of the individual constructs. For example, in the presence of the zE the −5.0 Kb promoter induces expression exclusively in the hematopoietic region (**Table 3**, **Figure 5A**). Since the enhancer element contains multiple Prep1-Pbx binding sites, it is possible that *prep1.1* is either auto-regulated, or controlled by one or more of the Prep1-homologous Meis proteins. Even though the expressiondata generated by embryos injection can depend on a variety of factors like the state of the chromatin at the site of integration, they, nevertheless, generate useful information in terms of “possible” activities. For example, our data show that the −5 Kb region has specific expression information even though we have been able to generate a transgenic fish harbouring with only 1.8 Kb of the promoter that recapitulates the constitutive expression of endogenous *prep1.1* in the zebrafish embryo. Indeed, the −5.0 and the −1.8 Kb constructs have a different expression pattern. Likewise the −1.8 Kb promoter-driven GFP expression is different in the transient injection vs. transgenic fish. Overall, since the −1.8 Kb GFP transgenic fish recapitulates the expression of the endogenous *prep1.1*, one would be inclined to conclude that this region contains all the necessary regulatory information. However, we do not dismiss the additional more 5′ sequences. First, the techniques employed do not have the necessary resolution to identify all cells in which the gene is expressed. Second, we have not yet performed regulatory studies on endogenous *prep1.1* expression. Regulatory elements, in addition to tissue-specific elements, might be present in the more upstream part of the promoter. Third, also the −5.0 Kb promoter activity is totally abolished by the EGR-1 mutation (**Figure 6**). Finally, sequences within the whole 5 Kb of the zebrafish regulatory sequences are partially concentrated (with about 40% identity) in the −5 to −3 Kb of the human sequences. We conclude that the present data highlight the complexity of the zebrafish *prep1.1* promoter, the existence of negative and positive regulatory elements and identify a 1.8 Kb fragment that contains the minimal regulatory region of *prep1.1* (a scheme of the promoter is reproduced in **Figure 9**). Appropriate constructs carrying the minimal zebrafish promoter and the human (or mouse) sequences may be also of help to better dissect the human/mouse promoter elements.

It is interesting that the mutation of the EGR-1 site close to the TSS totally abolishes the *in vivo* activity of the injected -5.0*prep1.1*mut:GFP construct. Computer-assisted analysis of the zebrafish regulatory region obviously identifies a myriad of potential binding sites. However, it is interesting to notice that an EGR-1 binding site is present at the same position in human and zebrafish, very close to the TSS. As the TSS in mouse is not known precisely, we cannot align mouse sequence with human and zebrafish. However, the same EGR-1site is present in the mouse at approximately the same position. The essential requirement of the EGR-1 site in *prep1.1* expression is interesting because EGR-1 factor is required for hematopoietic stem cells proliferation [20]. The presence of the same site in mouse and man suggests that EGR-1 may be an essential activator of the *Prep1* promoter also in these species. Indeed, *Prep1* is expressed in the hematopoietic stem cell region of the mouse [13], and the *Prep1* hypomorphic mice display an HSC-deficient phenotype [13]. It is therefore possible, that at least part of the *Egr-1* hematopoietic phenotype [20] may be due to the lack of its effect on *Prep1*.

Our work has also identified an intronic element that in transfection and injection assays has enhancer activity that might be tissue-specific. Interestingly, the 5′ region of this fragment contains Prep-Pbx and Hox-Pbx binding sites (**Table 2**). This suggests that expression of *prep1.1* is either auto-regulated, or regulated by one of its partners or paralogs (Pbx or Meis) whose dimers can bind the same sequences. The availability of the transgenic −1.8 Kb prep1.1-GFP may be of use to elucidate this point.

In conclusion, the analysis of the zebrafish *prep1.1* promoter has provided partial but important information in zebrafish and provided several clues on the mechanism of expression of *Prep1* also in mammals. In zebrafish, the single mammalian *Prep1* gene is divided in *prep1.1* and *prep1.2.* While the expression of the former is not regulated by retinoic acid, *prep1.2* is induced by retinoic acid [15]. In the transgenic -1.8Kbprep1.1:GFP fish, the expression of GFP is however not affected by retinoic acid (data not shown).

## Materials and Methods

### Cell culture and transient transfection assay

HeLa cells [2], maintained in DMEM medium containing 2 mM L-glutamine, 100 µg/ml penicillin, 100 µg/ml streptomycin and 10% fetal bovine serum, were used for transfection assays at 37°C in 5% CO_2_. Trasfection assays were carried out in 6-well FuGENE (Invitrogen) culture plates according to the manufacturer's instructions. HeLa cells were incubated in 2 ml of complete DMEM medium. When the cells were approximately 50% confluent (24 hrs after plating), they were overlaid with plasmid DNA/FuGENE complexes (2 µg of total plasmid per well consisting of 1 µg of *pGL3* vector promoter constructs plus 1 µg of pEGFP) in a total volume of 1 ml Optimem medium and incubated at 37°C. The medium was replaced after 16 hrs with complete medium and cells incubated for an additional 42 hrs. Cells were then washed with phosphate buffered saline (PBS, 137 mM NaCl, 2.7 mM KCl, 4.3 mM Na_2_HPO4•7H_2_O, 1.4 mM KH_2_PO_4_, pH 7.3) and harvested in 400 µl of 1X lysis buffer. Cellular debris were removed by centrifugation at 12000 g for 5 min. To determine luciferase activity, 5 µl of lysate supernatant was mixed with 25 µl of luciferase substrate (Promega Inc.) and luminescence measured with a MLX Microtiter® plate Luminometer. To correct for variation in trasfection efficiency, GFP activity was also determined using flow cytometry. Data are expressed as relative luciferase activity, defined as luciferase activity normalized to the luciferase of the *pGL3-Basic* vector control and GFP. A *pGL3-control-promoter* (i.e. an SV40 promoter) vector was included in all assays. At least three replicates were included in each sample group, and experiments were repeated three times.

### Determination of the human *Prep1* Transcription Start Site (TSS)

Primers used for primer extension were labeled with T4 Polinucleotide kinase and (32P)-γ-ATP at 37 C for 30 min, followed by heat inactivation at 92°C for 2 min and purified with Quick Spin colums G-25 (Boehringer). A dephosphorylated ϕ X174 HinfI DNA (Promega) was also labeled and used as a marker. 50 µg of total RNA from exponentially growing Hela cells were mixed with labeled primers, ethanol-precipitated, resuspended in Hybridization Solution (40 mM Pipes pH 6,4; 1 mM EDTA; 0,4M NaCl; 80% formamide) and denatured for 10 min at 85°C. Annealing of denatured RNA and primer was performed at 37°C overnight following by ethanol-precipitation. Reverse Transcriptase reactions were performed using Superscript II (Invitrogen) at 45°C for 90 min and reactions were stopped at 70°C for 15 min, treated with DNase free Ribonuclease for 30 min at 37 C and purified on colums (Qiagen). Samples were denatured 5 min at 95 C and resolved in a 6–8% acrylamide-urea gels. Dried gels were exposed at –80°C using autoradiographic films.

### Determination of the zebrafish *prep1.1* TSS

We searched for the *prep1.1* TSS region through a RT-PCR based strategy. Total RNA was extracted from hundred zebrafish embryos, at different developmental stage, using TRIzol, purified with DNaseI and quantified by agarose-gel electrophoresis. mRNA was then retrotranscribed using SuperScriptIII kit (Invitrogen) and amplified using oligos specific for the *prep1.1* 5′-UTR region, reported in **Table 1**.

### DNA constructs for transfection and injection assays

Luciferase reporter constructs were prepared from the zebrafish *prep1.1* promoter and human *Prep1* promoter including or not the first part of the intron-1 sequence. Fragments of different size (from the established TSS defined as +1) of human and zebrafish *Prep* genes, were used: 100 bp (from −1 to −100), 700 (from −1 to −700), 1800 (from −1 to −1800) and 5000 (from −1 to −5000) were cloned in the pGL3 vector. These clones were produced also in a version containing the first 563 bp of the zf intron 1. To generate these constructs by PCR, 5′ and 3′ primers with different restriction sites were designed and reported in **Table 4**. All DNA constructs were analyzed by restriction enzyme mapping and nucleotide sequencing.

**Table 4:** List of the oligonucleotides used to clone 5-untranscribed regions of *prep1.1*.

	Oligonucleotide
humanF100 bp	5′-cgaggcaaatggtttcgcccg-3′
humanF700 bp	5′-ctgcagtattgaaggccagtcagg-3′
humanF1800 bp	5′-ccaaagaatggctactccataggcag-3′
humanF5000 bp	5′-gcaattctcatgcctctgtgataccc-3′
humanR(for every construct)	5′-gcagagcctcccgccttatgac-3′
zebrafishF100 bp	5′-ggacagaacaccagagaactaattagc-3′
zebrafishF700 bp	5′-cttatgttacttgcgttaccctcaatactg-3′
zebrafishF1800 bp	5′-gcctttactgcatggcctgtgtacattttg-3′
zebrafishF5000 bp	5′-gcattatgcagtgagtctagctccac-3′
zebrafishR(for every construct)	5′-cggggaggagaagtgacgtgatgc-3′
humanFint	5′-gtaagctgtcactcaaccgctcc-3′
humanRint	5′-catgcgggccggtgtgattg-3′
zebrafishFint	5′-gtaagctgtcactcaaactcgtc-3′
zebrafishRint	5′-gggagtaaatgtagcagcggtctc-3′

F stands for forward; R for reverse.

### Transient expression in embryos and generation of transgenic of *prep1.1*-driven GFP zebrafish

Work with zebrafish does not require institutional ethics permission and was carried out under EU regulations for animal experimentation. The same constructs used for *in vitro* experiments on zebrafish promoter were used also for the *in vivo* experiments.

Zebrafish were maintained and crossed according to standard methods [21]. For the generation of the gene trap lines, we employed a plasmid [22], containing GFP and Tol2 elements, using different restriction enzymes depending on the fragment used. Embryos at the 1- to 2-cell stage were injected with 2 nl of a mixture containing 35 ng/µl of the circular *XigDpLynker* plasmid and 35 ng/µl of T2 transposase mRNA. Injected embryos were transferred to Petri dishes with E3 medium and tested for transient GFP expression at 24hpf using a Nikon stereomicroscope with a fluorescent light source. Embryos expressing GFP were collected and raised to adults as described [21]. Founders (F0) with germline transmission of the *1.8prep1.1:GFP* transgene were identified by pair-crossing injected zebrafish after they reached adulthood, and their progeny was screened for GFP expression at 24 hpf. GFP-positive Progeny (F1 generation) was raised to adulthood to establish the transgenic line: *Tg(-1.8prep1.1:GFP)*.

### Construction of the EGR-1 binding site mutant DNA

To mutagenize zebrafish EGR-1 DNA binding site we used the reverse oligonucleotide 5′-agtccgggacaatttacttttaaaac-3′, the forward oligonucleotide 5′-ctcccataacggggagtgtttttt-3′, the plasmid containing the 5.0 Kb *pknox1.1* promoter as template and *Pfu* DNA polymerase. Copies of the full plasmid in which the EGR-1 binding site was changed from TAACGGGGAGTG to TAACGTTGAGTG were generated by PCR. After *DNPI-*mediated degradation of the plasmid template, we circularized the phosphorilated PCR product by self-ligation and used it to transform competent bacteria. All DNA from tranformed colonies were purified, verified by nucleotide sequencing and used for the microinjection experiments in the zebrafish embryos.

### Whole-mount *in situ* hybridization

Embryos were fixed in 4% buffered *p*-formaldehyde. RNA in situ hybridizations were performed essentially as reported [23]. Digoxigenin and fluorescein-labelled antisense probes were synthesized from cDNAs of *prep1.1* (RZPD clone MPMGp609C2025Q8) and *gata1* [24]. Results of the hybridizations were analyzed statistically using Chi-square analysis.

### Immunohistochemistry

Zebrafish embryos, at different developmental stages, and 50 µm thick sagittal sections of 6 weeks juveniles, obtained with a vibratome, were fixed in 4% PFA and processed for immunohistochemistry as previously described [25]. The labeled sections were observed under a Leica compound microscope, equipped with fluorescent lamp and GFP filter. In the experiment of double staining (whole mount in situ hybridization combined with immunohistochemistry) the GFP expression was revealed with DAB and the embryos were mounted in glycerol and viewed with a Leica stereomicroscope.

## Supporting Information

Figure S1 (TIF)

Figure S2 Homology in the transcription start site region of the human and mouse sequence.
(TIF)
