# Correction: Characterization of the Regulatory Region of the Zebrafish *Prep1.1* Gene: Analogies to the Promoter of the Human *PREP1*

**DOI:** 10.1371/annotation/6de90ec9-0314-40ec-b634-27373373aabc

**Published:** 2011-04-05

**Authors:** Elisa Bernardi, Gianluca Deflorian, Federica Pezzinenti, Victor M. Diaz, Marina Mione, Francesco Blasi

The third author's name is misspelled. The correct spelling is Federica Pezzimenti. The correct citation is: Bernardi E, Deflorian G, Pezzimenti F, Diaz VM, Mione M, et al. (2010) Characterization of the Regulatory Region of the Zebrafish Prep1.1 Gene: Analogies to the Promoter of the Human PREP1. PLoS ONE 5(12): e15047. doi:10.1371/journal.pone.0015047

