# Supplementary figures and images for: Characterization of the Regulatory Region of the Zebrafish Prep1.1 Gene: Analogies to the Promoter of the Human PREP1
Source: PLoS One. 2010 Dec 22;5(12):e15047. doi: 10.1371/journal.pone.0015047 (PMC3008670; doi:10.1371/journal.pone.0015047)

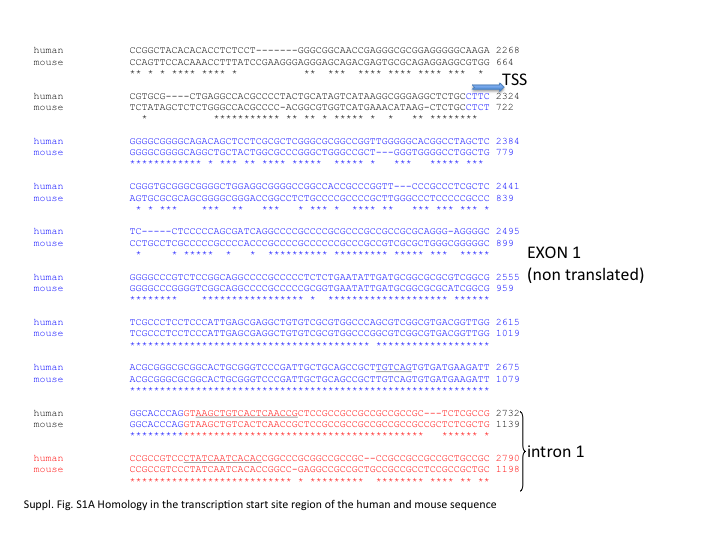

Supplement: Figure S1 — (TIF) [file pone.0015047.s001.tif]

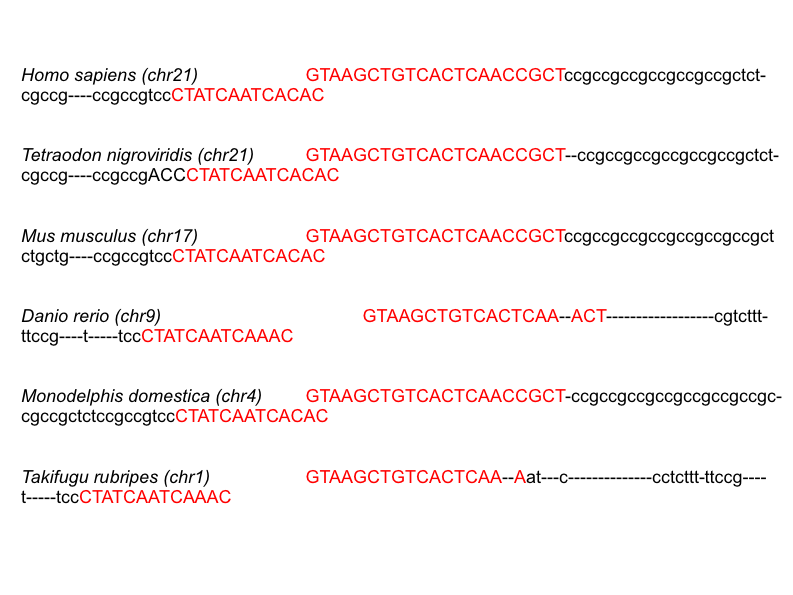

Supplement: Figure S2 — Homology in the transcription start site region of the human and mouse sequence. (TIF) [file pone.0015047.s002.tif]
